# Supplementary figures and images for: What lurks in the dark? An innovative framework for studying diverse wild insect microbiota
Source: Microbiome. 2025 Aug 12;13:186. doi: 10.1186/s40168-025-02169-9 (PMC12341219; doi:10.1186/s40168-025-02169-9)

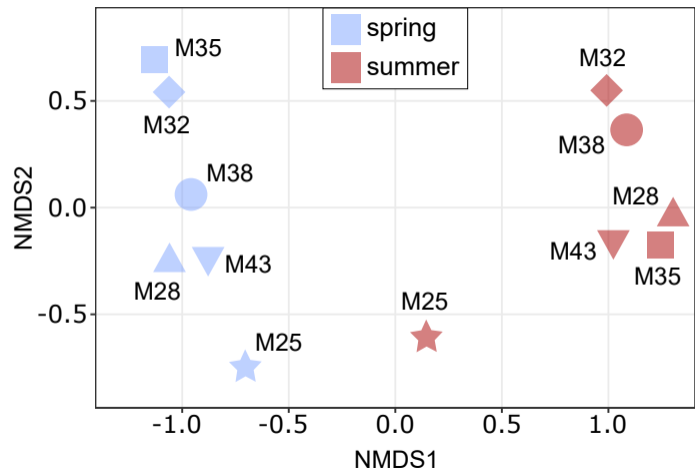

Supplement: Supplementary file 2 — Supplementary Material 1. [file 40168_2025_2169_MOESM1_ESM.pdf]

16S rRNA OTUs

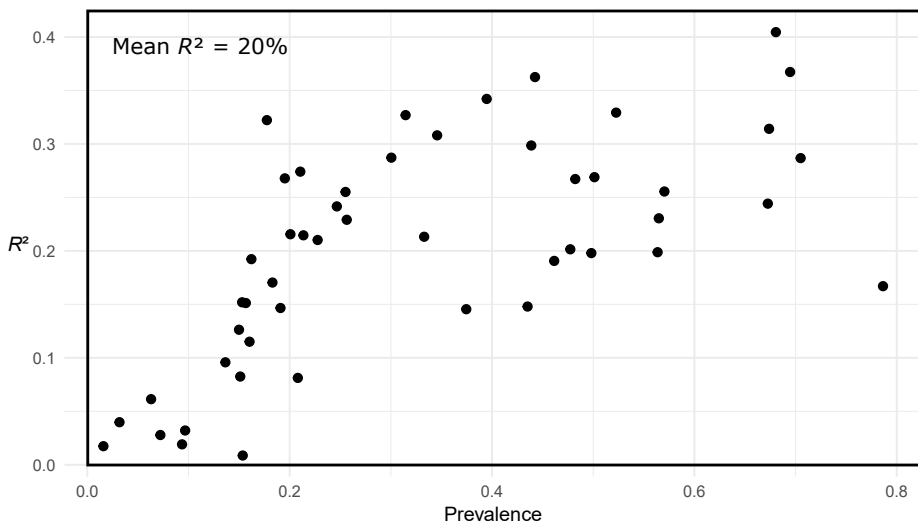

16S rRNA ZOTUs

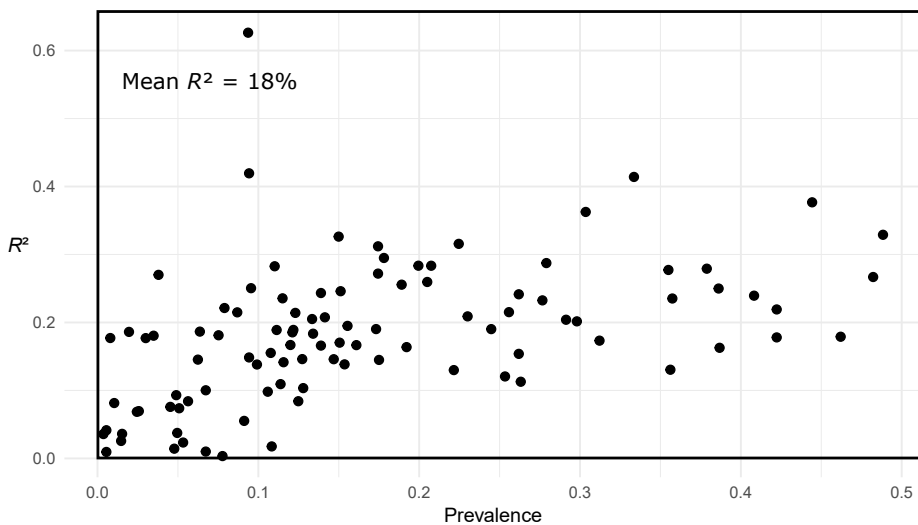

COI *Wolbachia* ZOTUs

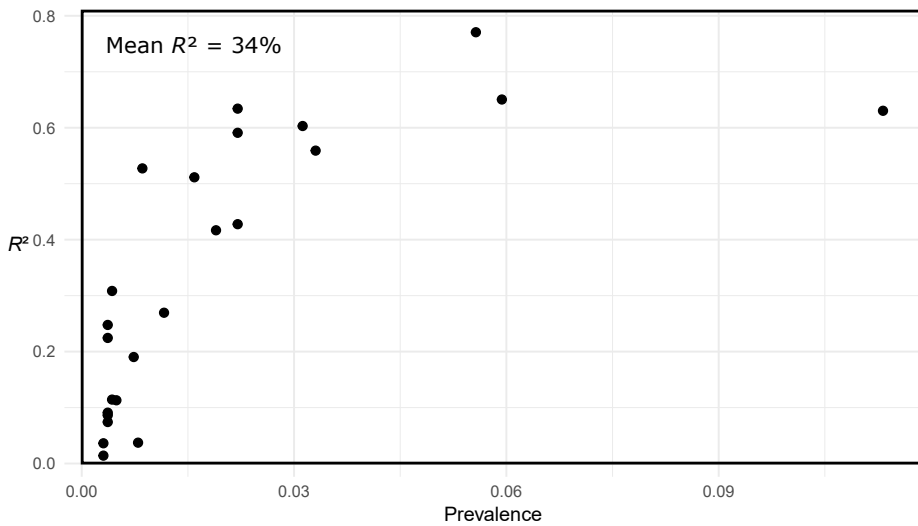

Supplement: Supplementary file 5 — Supplementary Material 4. [file 40168_2025_2169_MOESM4_ESM.pdf]
