# Supplementary material for: What lurks in the dark? An innovative framework for studying diverse wild insect microbiota
Source: Microbiome. 2025 Aug 12;13:186. doi: 10.1186/s40168-025-02169-9 (PMC12341219; doi:10.1186/s40168-025-02169-9)
Supplement: Supplementary file 3 — Supplementary Material 2. [file 40168_2025_2169_MOESM2_ESM.pdf]

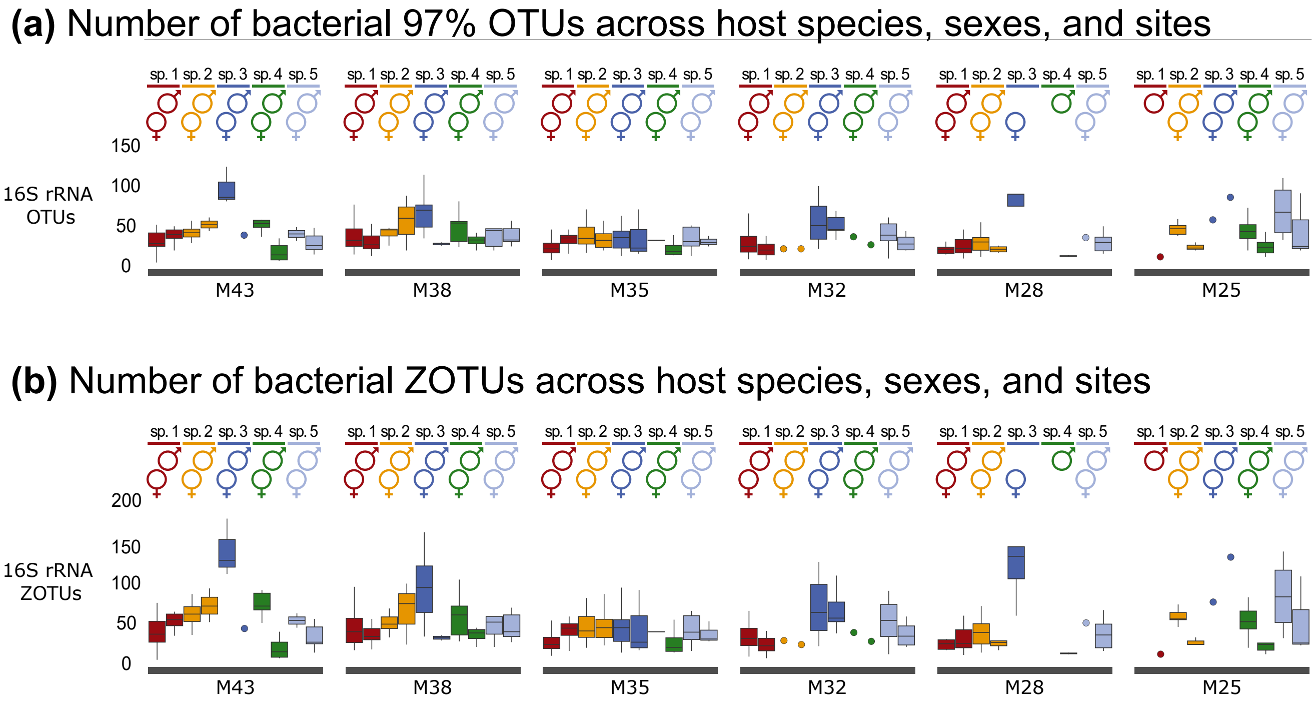

(a) Number of bacterial 97% OTUs across host species, sexes, and sites
sp. 1 sp. 2 sp. 3 sp. 4 sp. 5
16S rRNA OTUs
150
100
50
0
M43
M38
M35
M32
M28
M25
(b) Number of bacterial ZOTUs across host species, sexes, and sites
sp. 1 sp. 2 sp. 3 sp. 4 sp. 5
16S rRNA ZOTUs
200
150
100
50
0
M43
M38
M35
M32
M28
M25
