# Supplementary material for: What lurks in the dark? An innovative framework for studying diverse wild insect microbiota
Source: Microbiome. 2025 Aug 12;13:186. doi: 10.1186/s40168-025-02169-9 (PMC12341219; doi:10.1186/s40168-025-02169-9)
Supplement: Supplementary file 4 — Supplementary Material 3. [file 40168_2025_2169_MOESM3_ESM.pdf]

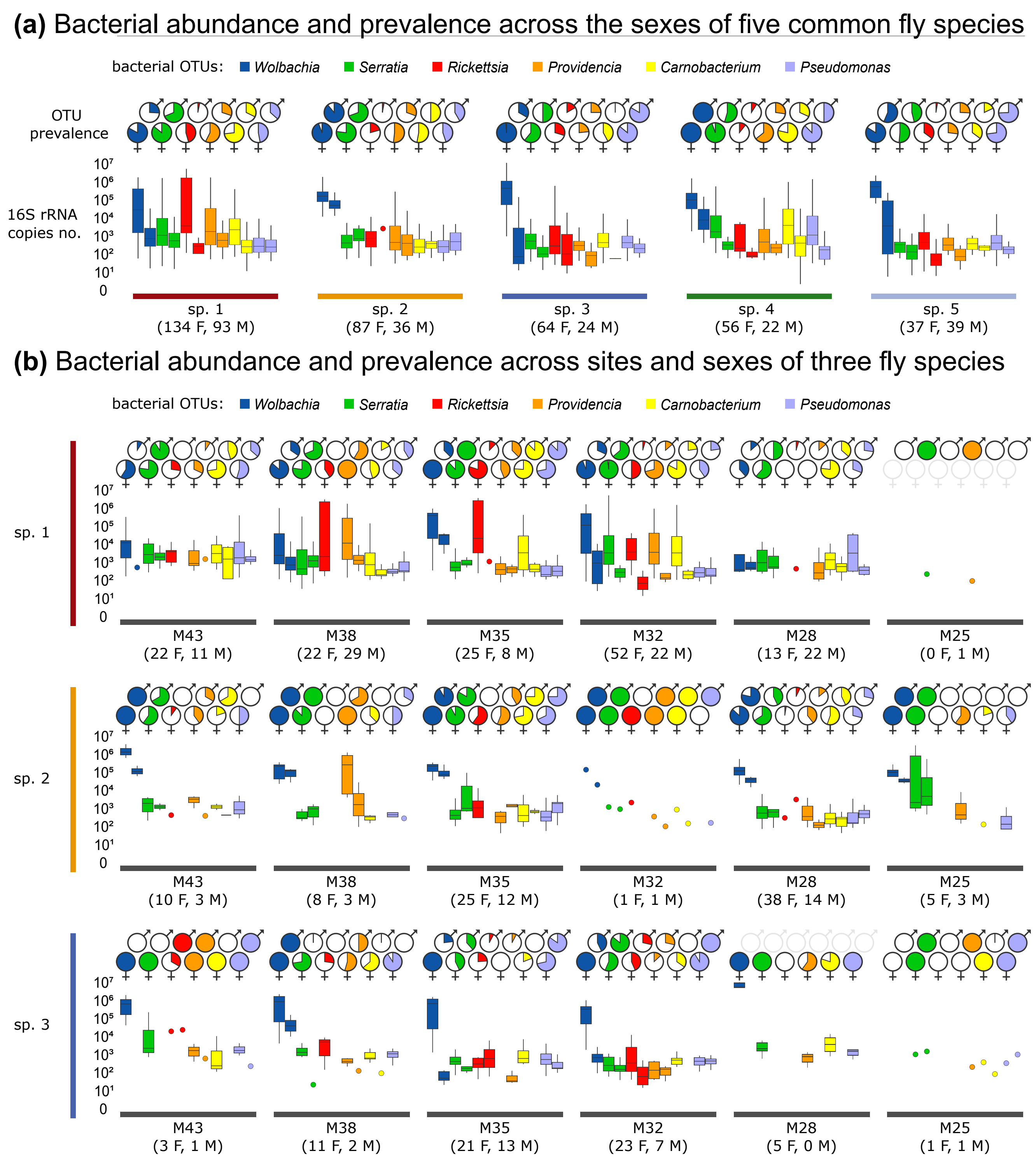
(a) Bacterial abundance and prevalence across the sexes of five common fly species
bacterial OTUs: Wolbachia Serratia Rickettsia Providencia Carnobacterium Pseudomonas
OTU prevalence
16S rRNA copies no.
10⁷ 10⁶ 10⁵ 10⁴ 10³ 10² 10¹ 0
sp. 1 (134 F, 93 M)
sp. 2 (87 F, 36 M)
sp. 3 (64 F, 24 M)
sp. 4 (56 F, 22 M)
sp. 5 (37 F, 39 M)
(b) Bacterial abundance and prevalence across sites and sexes of three fly species
bacterial OTUs: Wolbachia Serratia Rickettsia Providencia Carnobacterium Pseudomonas
sp. 1
M43 (22 F, 11 M)
M38 (22 F, 29 M)
M35 (25 F, 8 M)
M32 (52 F, 22 M)
M28 (13 F, 22 M)
M25 (0 F, 1 M)
sp. 2
M43 (10 F, 3 M)
M38 (8 F, 3 M)
M35 (25 F, 12 M)
M32 (1 F, 1 M)
M28 (38 F, 14 M)
M25 (5 F, 3 M)
sp. 3
M43 (3 F, 1 M)
M38 (11 F, 2 M)
M35 (21 F, 13 M)
M32 (23 F, 7 M)
M28 (5 F, 0 M)
M25 (1 F, 1 M)
