# Supplementary material for: What lurks in the dark? An innovative framework for studying diverse wild insect microbiota
Source: Microbiome. 2025 Aug 12;13:186. doi: 10.1186/s40168-025-02169-9 (PMC12341219; doi:10.1186/s40168-025-02169-9)
Supplement: Supplementary file 6 — Supplementary Material 5. [file 40168_2025_2169_MOESM5_ESM.pdf]

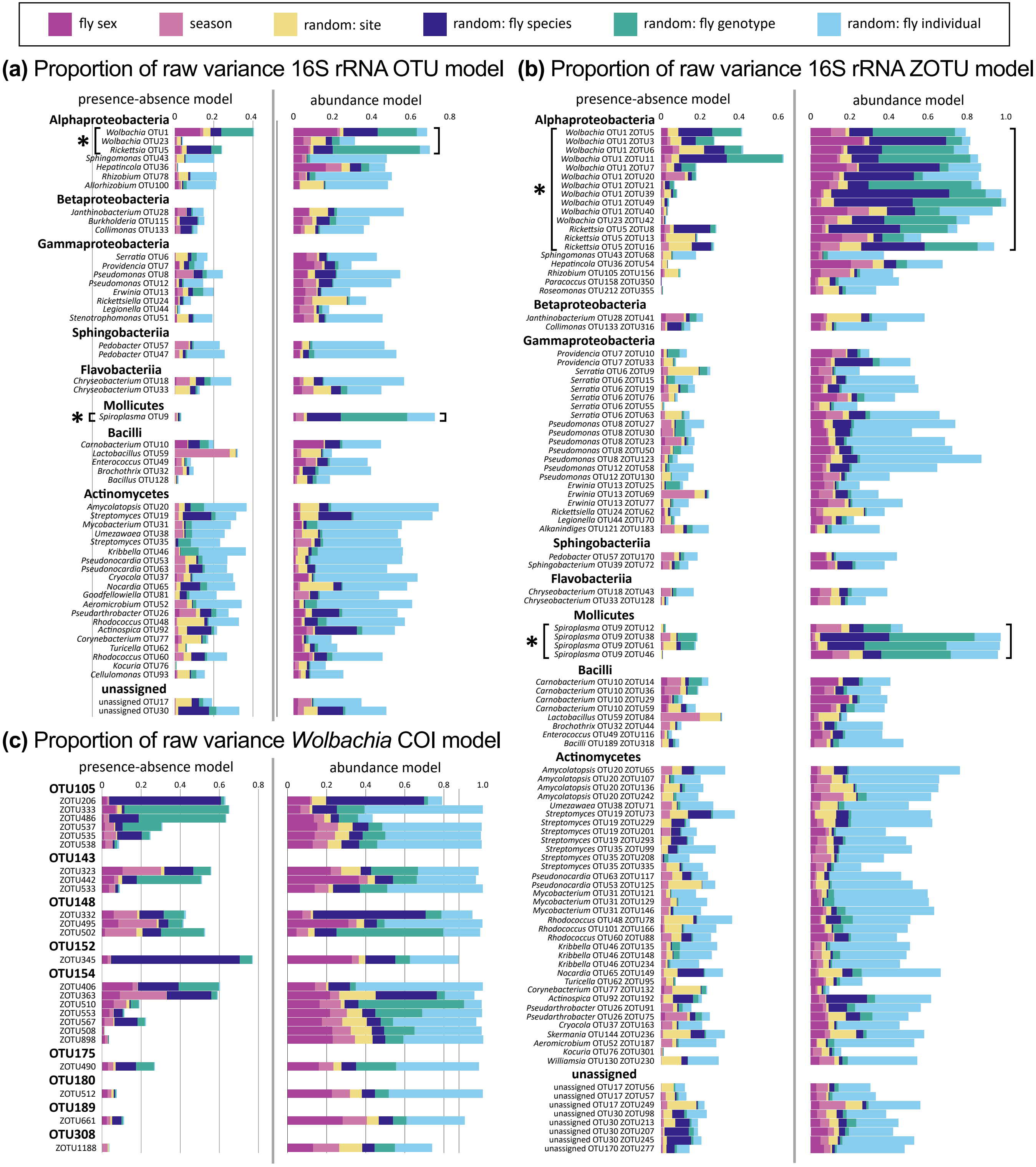

fly sex
season
random: site
random: fly species
random: fly genotype
random: fly individual
(a) Proportion of raw variance 16S rRNA OTU model
presence-absence model
0
0.2
0.4
abundance model
0
0.2
0.4
0.6
0.8
Alphaproteobacteria
*
Wolbachia OTU1
Wolbachia OTU23
Rickettsia OTU5
Sphingomonas OTU43
Hepatincola OTU36
Rhizobium OTU78
Allorhizobium OTU100
Betaproteobacteria
Janthinobacterium OTU28
Burkholderia OTU115
Collimonas OTU133
Gammaproteobacteria
Serratia OTU6
Providencia OTU7
Pseudomonas OTU8
Pseudomonas OTU12
Erwinia OTU13
Rickettsiella OTU24
Legionella OTU44
Stenotrophomonas OTU51
Sphingobacteriia
Pedobacter OTU57
Pedobacter OTU47
Flavobacteriia
Chryseobacterium OTU18
Chryseobacterium OTU33
Mollicutes
*
Spiroplasma OTU9
Bacilli
Carnobacterium OTU10
Lactobacillus OTU59
Enterococcus OTU49
Brochothrix OTU32
Bacillus OTU128
Actinomycetes
Amycolatopsis OTU20
Streptomyces OTU19
Mycobacterium OTU31
Umezawaea OTU38
Streptomyces OTU35
Kribbella OTU46
Pseudonocardia OTU53
Pseudonocardia OTU63
Cryocola OTU37
Nocardia OTU65
Goodfellowiella OTU81
Aeromicrobium OTU52
Pseudarthrobacter OTU26
Rhodococcus OTU48
Actinospica OTU92
Corynebacterium OTU77
Turicella OTU62
Rhodococcus OTU60
Kocuria OTU76
Cellulomonas OTU93
unassigned
unassigned OTU17
unassigned OTU30
(b) Proportion of raw variance 16S rRNA ZOTU model
presence-absence model
0
0.2
0.4
0.6
abundance model
0
0.2
0.4
0.6
0.8
1.0
Alphaproteobacteria
*
Wolbachia OTU1 ZOTU5
Wolbachia OTU1 ZOTU3
Wolbachia OTU1 ZOTU6
Wolbachia OTU1 ZOTU11
Wolbachia OTU1 ZOTU7
Wolbachia OTU1 ZOTU20
Wolbachia OTU1 ZOTU21
Wolbachia OTU1 ZOTU39
Wolbachia OTU1 ZOTU49
Wolbachia OTU1 ZOTU40
Wolbachia OTU23 ZOTU42
Rickettsia OTU5 ZOTU8
Rickettsia OTU5 ZOTU13
Rickettsia OTU5 ZOTU16
Sphingomonas OTU43 ZOTU68
Hepatincola OTU36 ZOTU54
Rhizobium OTU105 ZOTU156
Paracoccus OTU158 ZOTU350
Roseomonas OTU212 ZOTU355
Betaproteobacteria
Janthinobacterium OTU28 ZOTU41
Collimonas OTU133 ZOTU316
Gammaproteobacteria
Providencia OTU7 ZOTU10
Providencia OTU7 ZOTU33
Serratia OTU6 ZOTU9
Serratia OTU6 ZOTU15
Serratia OTU6 ZOTU19
Serratia OTU6 ZOTU76
Serratia OTU6 ZOTU55
Serratia OTU6 ZOTU63
Pseudomonas OTU8 ZOTU27
Pseudomonas OTU8 ZOTU30
Pseudomonas OTU8 ZOTU23
Pseudomonas OTU8 ZOTU50
Pseudomonas OTU8 ZOTU123
Pseudomonas OTU12 ZOTU58
Pseudomonas OTU12 ZOTU130
Erwinia OTU13 ZOTU25
Erwinia OTU13 ZOTU69
Erwinia OTU13 ZOTU77
Rickettsiella OTU24 ZOTU62
Legionella OTU44 ZOTU70
Alkanindiges OTU121 ZOTU183
Sphingobacteriia
Pedobacter OTU57 ZOTU170
Sphingobacterium OTU39 ZOTU72
Flavobacteriia
Chryseobacterium OTU18 ZOTU43
Chryseobacterium OTU33 ZOTU128
Mollicutes
*
Spiroplasma OTU9 ZOTU12
Spiroplasma OTU9 ZOTU38
Spiroplasma OTU9 ZOTU61
Spiroplasma OTU9 ZOTU46
Bacilli
Carnobacterium OTU10 ZOTU14
Carnobacterium OTU10 ZOTU36
Carnobacterium OTU10 ZOTU29
Carnobacterium OTU10 ZOTU59
Lactobacillus OTU59 ZOTU84
Brochothrix OTU32 ZOTU44
Enterococcus OTU49 ZOTU116
Bacilli OTU189 ZOTU318
Actinomycetes
Amycolatopsis OTU20 ZOTU65
Amycolatopsis OTU20 ZOTU107
Amycolatopsis OTU20 ZOTU136
Amycolatopsis OTU20 ZOTU242
Umezawaea OTU38 ZOTU71
Streptomyces OTU19 ZOTU73
Streptomyces OTU19 ZOTU229
Streptomyces OTU19 ZOTU201
Streptomyces OTU19 ZOTU293
Streptomyces OTU35 ZOTU99
Streptomyces OTU35 ZOTU208
Streptomyces OTU35 ZOTU335
Pseudonocardia OTU63 ZOTU117
Pseudonocardia OTU53 ZOTU125
Mycobacterium OTU31 ZOTU121
Mycobacterium OTU31 ZOTU129
Mycobacterium OTU31 ZOTU146
Rhodococcus OTU48 ZOTU78
Rhodococcus OTU101 ZOTU166
Rhodococcus OTU60 ZOTU88
Kribbella OTU46 ZOTU135
Kribbella OTU46 ZOTU148
Kribbella OTU46 ZOTU234
Nocardia OTU65 ZOTU149
Turicella OTU62 ZOTU95
Corynebacterium OTU77 ZOTU132
Actinospica OTU92 ZOTU192
Pseudarthrobacter OTU26 ZOTU91
Pseudarthrobacter OTU26 ZOTU75
Cryocola OTU37 ZOTU163
Skermania OTU144 ZOTU236
Aeromicrobium OTU52 ZOTU187
Kocuria OTU76 ZOTU301
Williamsia OTU130 ZOTU230
unassigned
unassigned OTU17 ZOTU56
unassigned OTU17 ZOTU57
unassigned OTU17 ZOTU249
unassigned OTU30 ZOTU98
unassigned OTU30 ZOTU213
unassigned OTU30 ZOTU207
unassigned OTU30 ZOTU245
unassigned OTU170 ZOTU277
(c) Proportion of raw variance Wolbachia COI model
presence-absence model
0
0.2
0.4
0.6
0.8
abundance model
0
0.2
0.4
0.6
0.8
1.0
OTU105
ZOTU206
ZOTU333
ZOTU486
ZOTU537
ZOTU535
ZOTU538
OTU143
ZOTU323
ZOTU442
ZOTU533
OTU148
ZOTU332
ZOTU495
ZOTU502
OTU152
ZOTU345
OTU154
ZOTU406
ZOTU363
ZOTU510
ZOTU553
ZOTU567
ZOTU508
ZOTU898
OTU175
ZOTU490
OTU180
ZOTU512
OTU189
ZOTU661
OTU308
ZOTU1188
