# Supplementary material for: What lurks in the dark? An innovative framework for studying diverse wild insect microbiota
Source: Microbiome. 2025 Aug 12;13:186. doi: 10.1186/s40168-025-02169-9 (PMC12341219; doi:10.1186/s40168-025-02169-9)
Supplement: Supplementary file 7 — Supplementary Material 6. [file 40168_2025_2169_MOESM6_ESM.pdf]

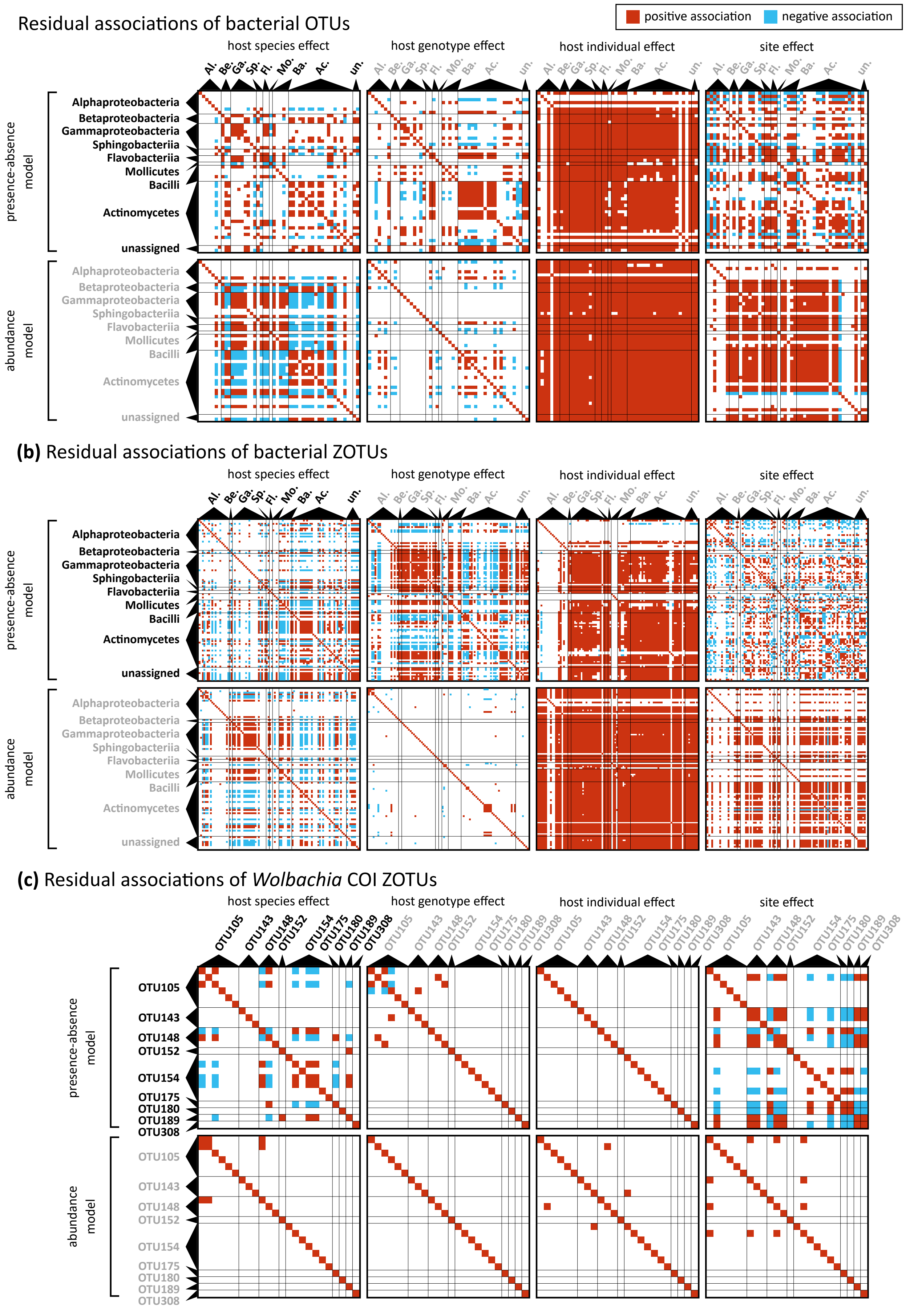

Residual associations of bacterial OTUs
positive association
negative association
host species effect
host genotype effect
host individual effect
site effect
Al.
Be.
Ga.
Sp.
Fl.
Mo.
Ba.
Ac.
un.
presence-absence model
abundance model
Alphaproteobacteria
Betaproteobacteria
Gammaproteobacteria
Sphingobacteriia
Flavobacteriia
Mollicutes
Bacilli
Actinomycetes
unassigned
(b) Residual associations of bacterial ZOTUs
(c) Residual associations of Wolbachia COI ZOTUs
OTU105
OTU143
OTU148
OTU152
OTU154
OTU175
OTU180
OTU189
OTU308
